# Antimony as a Programmable Element in Integrated Nanophotonics

**DOI:** 10.1021/acs.nanolett.1c04286

**Published:** 2022-04-22

**Authors:** Samarth Aggarwal, Tara Milne, Nikolaos Farmakidis, Johannes Feldmann, Xuan Li, Yu Shu, Zengguang Cheng, Martin Salinga, Wolfram HP Pernice, Harish Bhaskaran

**Affiliations:** † Department of Materials, University of Oxford, Parks Road, Oxford OX1 3PH, U.K.; ‡ State Key Laboratory of ASIC and System, School of Microelectronics, Fudan University, Shanghai 200433, China; § Institut für Materialphysik, Westfälische Wilhelms-Universität Münster, Wilhelm-Klemm-Straße 10, 48149, Münster, Germany; ∥ Kirchhoff-Institute for Physics, Heidelberg University, 69120 Heidelberg, Germany

**Keywords:** Phase change materials, Antimony, Ultrafast switching, Metallic glass, Femtosecond Processing

## Abstract

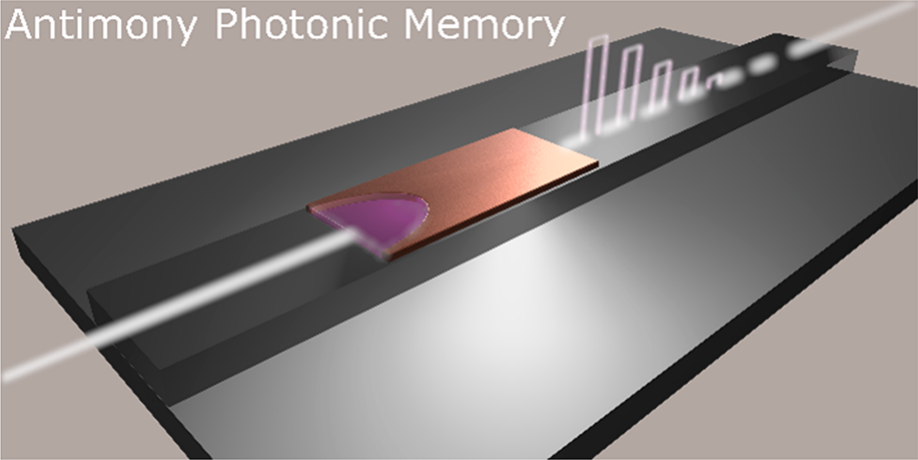

The use of nonlinear elements with memory as photonic computing components has seen a huge surge in interest in recent years with the rise of artificial intelligence and machine learning. A key component is the nonlinear element itself. A class of materials known as phase change materials has been extensively used to demonstrate the viability of such computing. However, such materials continue to have relatively slow switching speeds, and issues with cyclability related to phase segregation of phase change alloys. Here, using antimony (Sb) thin films with thicknesses less than 5 nm we demonstrate reversible, ultrafast switching on an integrated photonic platform with retention time of tens of seconds. We use subpicosecond pulses, the shortest used to switch such elements, to program seven distinct memory levels. This portends their use in ultrafast nanophotonic applications ranging from nanophotonic beam steerers to nanoscale integrated elements for photonic computing.

With ever-increasing demands for computing and electronics reaching a limit, all-photonic neuromorphic computing architectures based on nonlinear attenuating elements have gained prominence.^1−5^ Phase change materials are strong candidates, as they are not only effective attenuators but retain their state once programmed, providing a “non-volatile memory” element.^6−11^ Neuromorphic photonic computing using phase change materials offers faster computing speeds, particularly in throughput, but the performance is usually limited by the switching speeds of the phase change materials, which limits the training speeds achievable.^12,13^

Conventional phase change materials like Ge_2_Sb_2_Te_5_ (GST) and Ag_3_In_4_Sb_76_Te_17_ (AIST) have shown to be effective for many applications in photonic computing.^14^ However, the shortcomings of these materials are two-fold. The first drawback of these materials is their switching speed. This is determined by their crystallization speed, which is typically of the order of 5–100 ns,^10,11^ although speeds of less than a nanosecond in GST have been reported.^15^ A second drawback is the compositional integrity of these alloys; ternary and quaternary PCMs undergo phase separation^16−18^ over several switching cycles which limits their cyclability. Related to compositional integrity is the difficulty in fine-tuning the target composition to ensure the correct stoichiometry, control of this is crucial to ensure phase stability, and cycling endurance.^19^

The issue of compositional integrity can be mitigated by using a single element, however not all elements are active. Elemental Sb at nanoscale dimensions was known to have different electrical conductance in amorphous and crystalline state,^20^ but its applications as a nonlinear optical material were not fully explored. Recently, it was shown that nanoscale thin films of Sb can be switched electrically^21,22^ and optically.^23^ In this paper, we demonstrate the integration of nanoscale Sb films in integrated photonics and perform on chip switching using subpicosecond laser pulses which couple to Sb evanescently. This is the first instance of their use in integrated photonics, which sets the stage for their potential use in a range of applications ranging from computing to routing.

## Switching between Binary States

**Figure 1:**
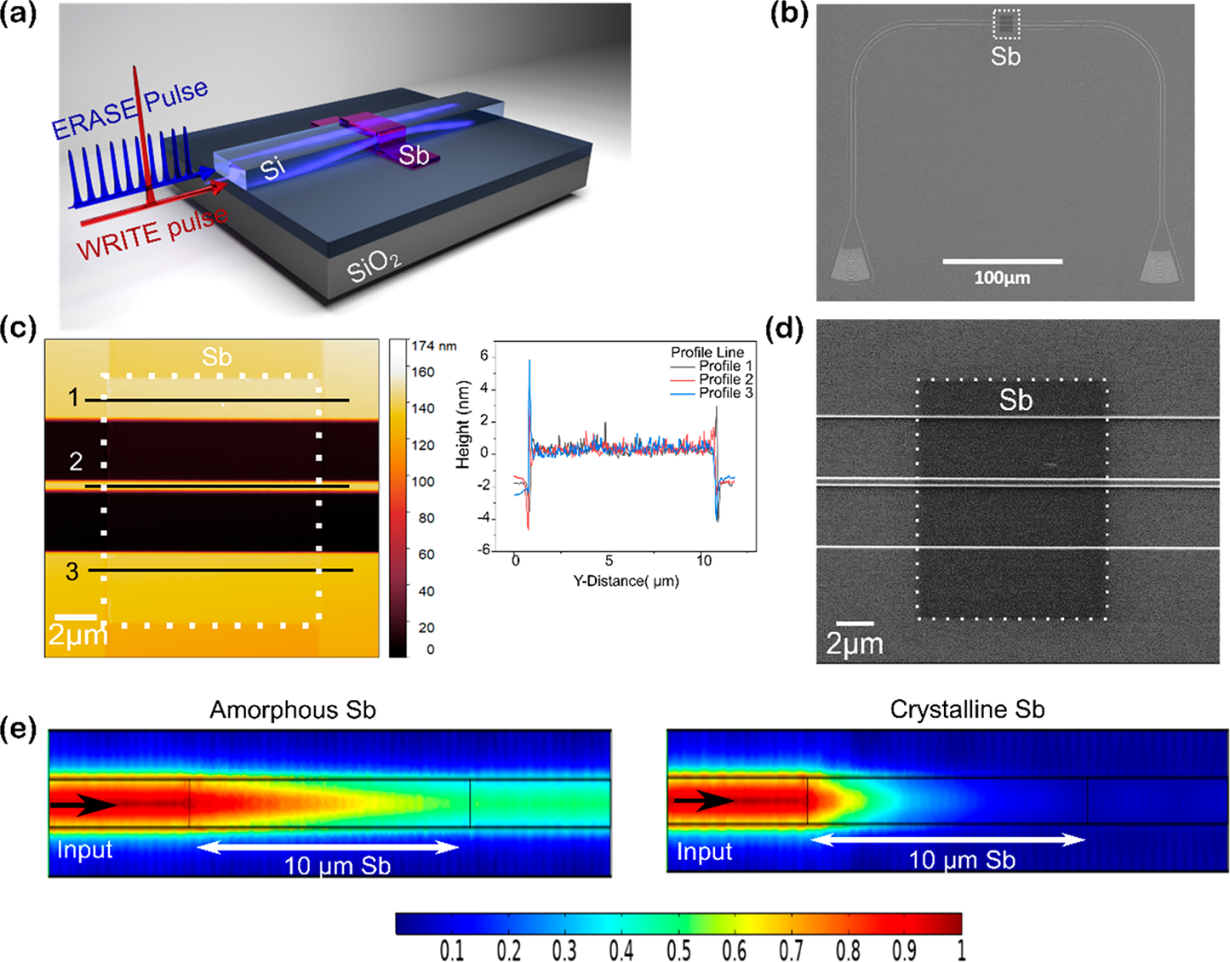
(a) Schematic of SOI waveguide with 3 nm Sb, an amorphization pulse (WRITE) or crystallization pulse (ERASE) is used to switch the PCM. (b) An SEM image showing the device structure, which includes a waveguide with grating couplers. White dotted box shows the PCM deposited on the waveguide. (c) AFM image of the region inside the box in (b) confirms the thickness of Sb to be 3 nm, (d) Zoomed-in SEM image showing device with Sb on the waveguide. (e) Frequency domain simulations using COMSOL showing the E-field distribution in silicon waveguide with 3 nm thick Sb. The colors represents the normalized (in the range 0–1) intensity of the electric field. Crystalline Sb is highly absorptive and thus results in lower transmission.

Our initial experiments were designed to investigate whether it was possible to switch antimony thin films on an integrated device. Figure 1a illustrates our device, which includes a partially etched SOI waveguide (450 nm wide on 220 nm SOI substrate with etch depth of 120 nm) operating at 1550 nm wavelength. It is known that it is important to confine the thickness of the Sb films below 10 nm to obtain nonvolatile switching behavior.^22,23^ Thus, Sb of thickness 3 nm is sputtered on top the waveguide, Figure 1c, following a patterning step to define the geometry of the Sb film; unlike with the use of phase change materials, here we use no capping layers. This is because, as we show in SI Section S4, there is no evidence of oxide layer formation on the Sb.

Using grating couplers, light is coupled in and out of our devices, as shown in an SEM image in Figure 1b,d. We use finite element modeling [COMSOL multiphysics frequency domain simulation] to calculate the degree of transmission through the waveguide for Sb in both the amorphous and crystalline states. The change in transmission arises due to the difference in absorption of the two states, as shown in Figure 1e. The light within the waveguide couples evanescently to the Sb film; the crystalline phase of nanometer-thin films of Sb absorbs more light when compared to its amorphous phase, as it has a higher attenuation coefficient *k* (*k* = 2.81 for crystalline and *k* = 0.61 for amorphous Sb at 1550 nm^23^). Therefore, we expect a change in the transmission of the waveguide which depends on the solid phase of Sb.

We initially crystallize Sb by annealing our device at 230 °C for 5 min in air on a hot plate; this allows the Sb to be in a more absorptive state, which enables more efficient near-field absorption of light. We use a fiber-coupled femtosecond laser (PriTel FFL-TW) in order to characterize the switching of these materials in integrated photonics. Light is coupled into and out of the device using grating couplers; details of the experimental setup are described in Figure S1. Using a single femtosecond pulse of high energy 194 ± 35pJ, we can switch Sb to its amorphous state (Write); we measure this as an increase in the transmission through the device using our probe laser, as this state is less absorptive. To crystallize (Erase) the material, we send 100 low energy pulses with an individual pulse of energy 45 ± 9pJ (total energy 4.5 nJ).

A lower energy pulse results in a smaller volume being above the recrystallization temperature. Therefore, multiple pulses are used to achieve reversible switching. In this work, we have not optimized the thermal design of the system nor have we determined the appropriate pulsing energy. As will be seen later in this manuscript, a different volume of the material requires experimental determination of the pulse sequence separately. We anticipate that with progress in this field through more research, these processes will be better understood similar to our current understanding in GST devices.^10^

**Figure 2:**
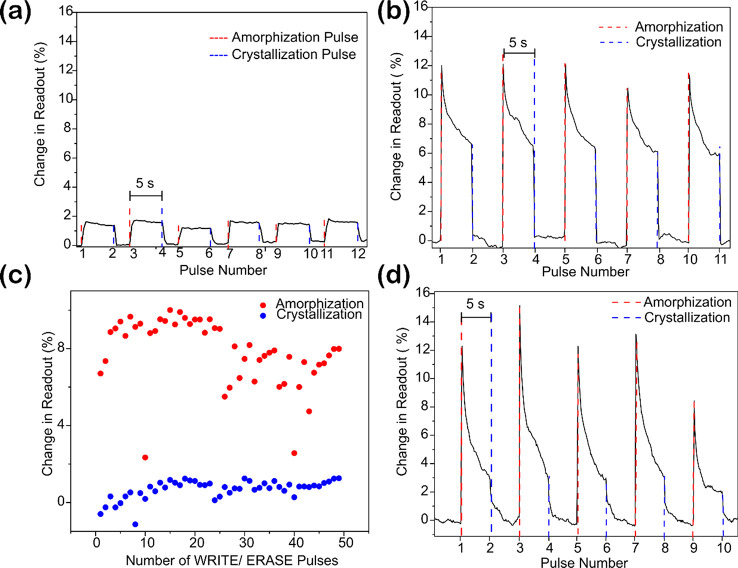
Binary switching on a waveguide, (a) Sb (length = 4 μm) on a waveguide switched using single, high energy (194 ± 35pJ) femtosecond pulse (800 fs) (amorphization pulse, red dashed vertical lines indicate this pulse). One hundred low energy (45 ± 9pJ) pulses (crystallization sequence, blue dashed lines indicate when this is sent) crystallize the sample. (b) Sb (length = 10 μm) switched between amorphous and crystalline phase as in (a). As expected, longer length results in higher contrast. (c) Multiple switching of device in (b). (d) Demonstration of both single-pulse amorphization and crystallization for the same device as in (b,c) by fine-tuning the pulse energies; a single pulse is enough for binary switching. The red dashed lines represent the pulse number at which an amorphization pulse of energy 91 ± 7pJ is sent, while the blue dashed lines represent a crystallization pulse of energy 59 ± 11pJ.

As shown in Figure 2a, we can switch the material between these two states for lengths of Sb down to 4 μm. For longer lengths of 10 μm, we achieve a higher (8%) contrast in the readout signal (Figure 2b). Here, contrast is defined as the percentage change in readout signal at time *t* = 5 s after amorphization pulse. For consistency of results, we use Sb of 10 μm length in the following experiments. Figure 2c shows that this process is repeatable for more than 50 cycles with a variability below 2%. This variability is attributed, in our case, to the variation in the pulse energies. In principle, a single element PCM should have superior cycling endurance; however, cyclability is a complex phenomenon. It is a function of not only the material but also delamination of thin films, determination of optimum switching pulse energies, and the use of capping layers. This work does not address those aspects. Future work must include more rigorous experimentation and analysis in order to verify how much the benefits of single element memories eventually increase cyclability.

To test the switching speed of our device, we perform time-resolved switching experiments as described in Figure S2. For a Write pulse, we obtain 500 MHz speed of operation using a single sub picosecond pulse.

We then demonstrate that a single low power femtosecond pulse crystallizes the material this is shown in Figure 2d. When the pulse energy is above the threshold (91 ± 7pJ) required to amorphize Sb we observe that the transmission changes (i.e., switching occurs). For powers below the amorphization threshold but above that required for crystallization (59 ± 11pJ), we observe crystallization. However, as seen in Figure 2d, using a single crystallization pulse does not form a stable memory level. Unlike multiple crystallization pulses, a single crystallization pulse results in an incomplete crystallization of the amorphous Sb volume. This intermediate crystalline level results in lower absorption of pulse energy and hence a lower contrast. We examine the effect of pulse energy, contrast, and stability of the memory operation in the later section.

## Programming Multiple Levels

**Figure 3:**
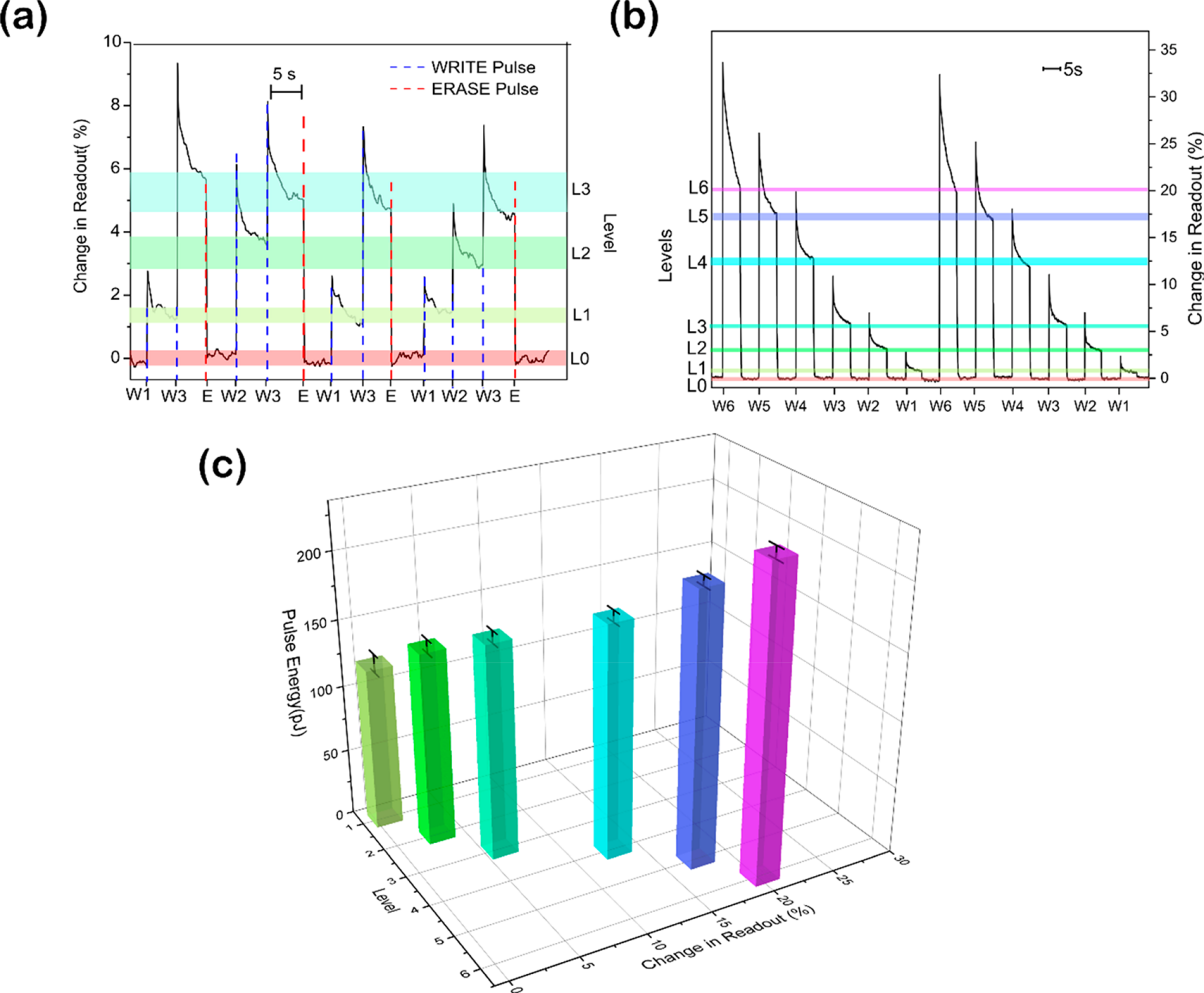
(a) Multilevel programming in Sb. Four distinct levels of transmission are programmed (termed as L0, L1, L2, and L3) using a single femtosecond (pulse width 800 fs) WRITE pulse (termed W1, W2, and W3) of energies 145 ± 6pJ, 150 ± 6pJ and 160 ± 5pJ, respectively. ERASE is achieved by sending 200 pulses of 100pJ femtosecond pulses. (b) Repeatedly reaching seven memory levels (L0–L6) by WRITE pulses W1–W6 (pulse energies 121 ± 7pJ, 144 ± 5pJ, 160 ± 5pJ, 174 ± 4pJ, 204 ± 4pJ, and 231 ± 4pJ respectively). (c) The pulse energies corresponding to each level.

Our next experiments seek to verify whether it is possible to reach intermediate transmission states by partially switching Sb. Analogous to photonic phase change memories,^24^ increasing the switching volume results in a higher contrast, as a larger area interacts with the near-field of the light transmitted within the waveguide. By changing the power used, the volume of Sb amorphized or crystallized can be controlled. Thus, by fine-tuning the pulse energy, we achieve multilevel programming as a function of discrete states of transmission. Figure 3a shows four distinguishable states that are programmed by varying the pulse energy. By sending a single pulse of fixed (800 fs) width and by varying the pulse energy (145 ± 6pJ, 150 ± 6pJ and 160 ± 5pJ), we reliably achieve three distinguishable states. We can achieve these states both arbitrarily and sequentially in ascending order, by sending a WRITE pulse corresponding to the desired level. For example, in Figure 3a a single pulse of 800 fs duration and 160pJ energy is sufficient to reach level L3 from either of the lower levels, L1 or L2. Once at a higher level, the lowest level L0, can be achieved by sending 100 low energy pulses of energy 101 ± 14pJ (ERASE).

Furthermore, we achieved seven distinguishable memory levels by increasing the WRITE pulse energies (from 121 ± 7pJ to 231 ± 4pJ), as shown in Figure 3b. We note that the variation in the pulse energies we report here in Figure 3 c is because of the noise of the energy-meter sensitivity (Ophir PD10-IR). To recrystallize, we send about 200 pulses in this higher contrast regime as compared to 100 pulses in the binary regime.

**Figure 4:**
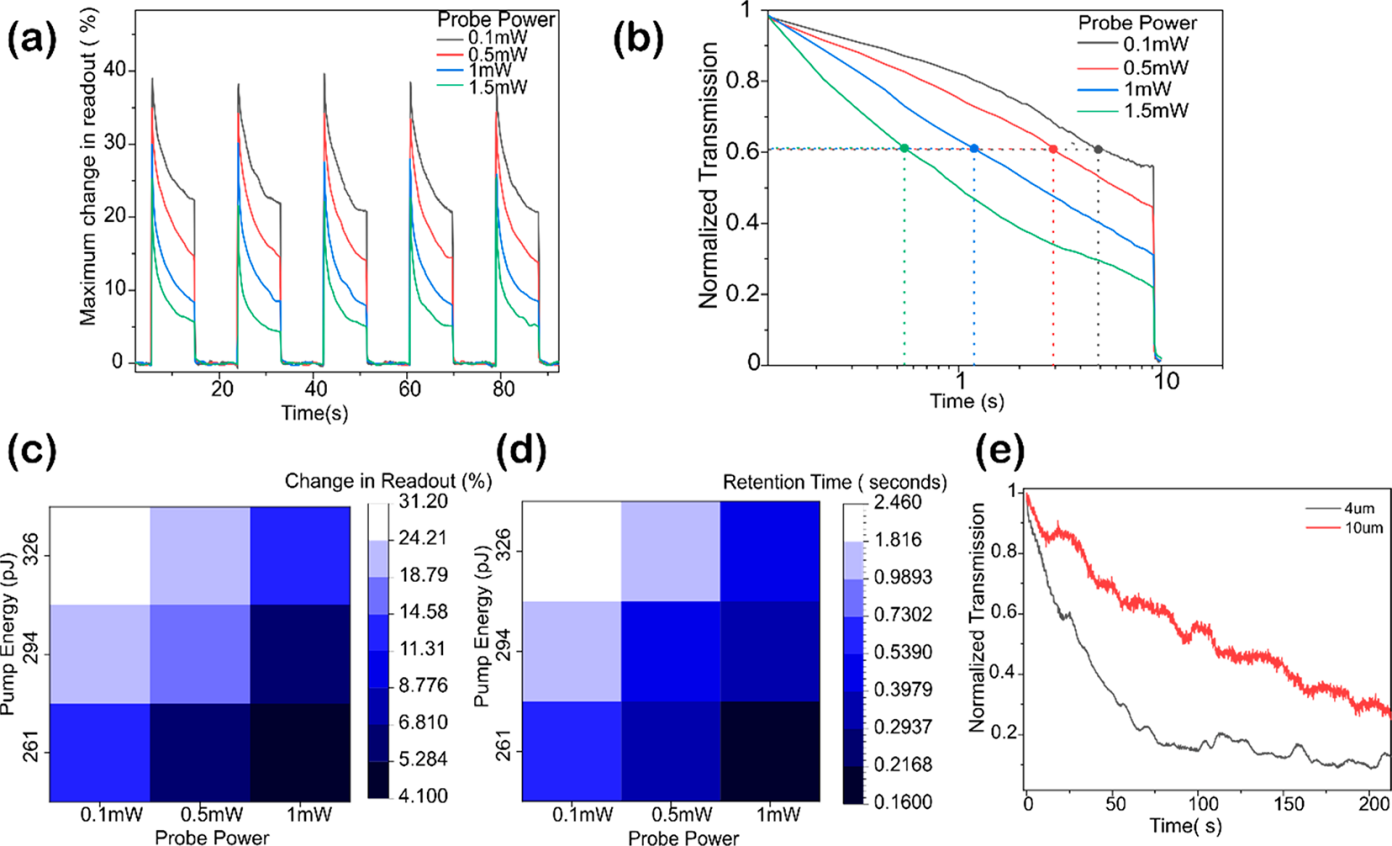
(a) Effect of the probe power on the maximum transmission achieved for a given pump power. An increase in probe power results in a decreased maximum change in the readout. (b) Effect of probe power on the retention time. We normalize individual plots between 0 and 1 corresponding to the lowest (crystalline) and the highest (amorphous) transmission. We plot the time required to reach 60% of the maximum change in readout. Increasing the probe power decreases the retention time. (c) Effect of both probe and pump power on the maximum readout contrast. A high pump and low probe power lead to the maximum change in the readout. (d) Effect of pump and probe power on retention time (time required to reach 90% of maximum change in readout). The volatility of Sb is controlled by varying either or both. (e) Long-term stability of 4 and 10 μm long amorphous Sb, switched from crystalline phase using 409 pJ a single femtosecond pulse. Normalization is done as in panel b.

We then investigate the effect of probe power on switching contrast and stability of memory states. We observe that the maximum contrast in the readout signal that can be achieved decreases with an increase in probe power (Figure 4a). As before, we use a single femtosecond pulse for amorphization and multiple low energy pulses for crystallization. In Figure 4b, we plot the normalized transmission and examine the time required for transmission to fall to 60% of its original value. We find an increase in probe power leads to a faster decay time. Similar behavior is observed in phase change materials like GST^25^ where an increase in Probe power results in recrystallization and therefore shorter retention time. However, unlike GST, where higher probe power leads to a higher contrast, we observe a lower contrast for Sb.

We further extend these experiments and observe the change in transmission and retention time on both pump and probe power. These experiments result in two important observations. First, as shown in Figure 4c, a higher pump power leads to an increased contrast in the readout and an increase in probe power results in decreased contrast. Second, there is a direct correlation between contrast and retention time. As represented in Figure 4c,d, higher contrast results in higher retention time. Here, we define retention time as time required to reach 90% of the maximum contrast. The samples with similar contrast, for example (probe power 0.1 mW and pump energy 294 pJ) and probe (0.5 mW with pump energy 326 pJ), have a similar retention time of 1.1 s. Understanding this direct correlation between contrast and retention time requires further analysis of recrystallization dynamics using in situ imaging techniques, beyond the scope of this work.

Finally, we look at the long-term switching stability of amorphous Sb for the devices in Figure 4e. We use a reference device without Sb (Figure S5), to account for stage drift and fluctuations in probe laser power over time. At time *t* = 0, we switch to amorphous phase using a single femtosecond pulse of 409 pJ and monitor the drop in transmission. The 4 and 10 μm long devices have a recrystallization 100 and 200 s, respectively. The intrinsic transient behavior can be used to implement leaky integrate and fire neurons^26,27^ and can be used for applications requiring combination of short- and long-term plasticity in neurons.^28^

We have further looked at the stability of intermediate memory levels, as described in SI Section S7. We observe stability of intermediate memory levels over 100 ms with a variation in readout of 2%. With a clock speed of only 1 GHz, millions of operations are possible, sufficient to carry out calculations such as tensor core operations^1−4^ and associative learning.^29^

## Conclusions

We have demonstrated for the first time that nanoscale thin films of antimony act as programmable elements on integrated waveguides. We switch the device with a single femtosecond pulse, which corresponds to a speed of 500 MHz. We demonstrate that Sb can be programmed beyond two states and achieve up to seven different discernible states, setting the stage for future improvements that could lead to higher-bit accuracy programming. The use of single element ultrathin films allows for potential thickness scaling, while also enabling long-term cyclability because of the lack of phase separations induced by repeated phase transitions. This opens up a plethora of applications in computing, where high cyclability and ultrafast speeds are required, such as efficient vector–matrix multiplications^1,3,4^ and accumulative processing^2,30−32^

## Methods

### Device Fabrication

The fabrication of waveguides is done using 220 nm Silicon on 3 μm oxide. The waveguides are patterned using electron-beam lithography using a positive photoresist. The pattern waveguide is then partially dry etched (etch depth 120 nm). The fabricated waveguides are subjected to the second round of patterning to open windows for Sb deposition using radio frequency (RF) sputtering (Nordiko sputtering systems). The thin film of Sb is deposited from a commercial sputtering target (Testbourne) at a deposition rate of 3.3 nm/min at 5 mTorr pressure in an argon environment with 30W RF power.

### Experimental Setup

We use a 40 MHz 1550 nm fiber coupled, femtosecond laser from Pritel (FFL-TW-60MhZ) with a pulse width of 800 fs. Using a home-built pulse picker, we select the number of pulses through the chip. The pulses are sent through a high peak power EDFA from Pritel (HPP-PMFA-21–10) to control energy for switching. Another CW laser is used for probing the transmission and observe the change in transmission levels after switching. An optical filter from Santec (OTF-320) is attached to the probe line before a 200 kHz photodetector (2011-FC-M) from Newport to isolate the pulse and probe signal. All of the photodetectors are connected to a computer using a DAQ to record data.

## Supporting Information Available

Section S1, experimental setup; Section S2, switching speed measurements; Section S3, Sb length dependence on contrast; Section S4, effect of capping layer; Section S5, pulse energy calculations; Section S6; submillisecond readout; S7, multilevel readout stability (PDF)
