# Supplementary material for: Antimony as a Programmable Element in Integrated Nanophotonics
Source: Nano Lett. 2022 Apr 22;22(9):3532–8. doi: 10.1021/acs.nanolett.1c04286 (PMC9101065; doi:10.1021/acs.nanolett.1c04286)
Supplement: Supplementary file 1 — nl1c04286_si_001.pdf [file nl1c04286_si_001.pdf]

# Antimony as a programmable element in integrated nanophotonics

*Samarth Aggarwal[1], Tara Milne[1], Nikolaos Farmakidis[1], Johannes Feldmann[1], Xuan Li[1], Yu Shu[1], Zengguang Cheng[1, 2], Martin Salinga[3], Wolfram HP Pernice[4] and Harish Bhaskaran[1]**

[1]Department of Materials, University of Oxford, Parks Road, Oxford OX1 3PH, UK

[2]State Key Laboratory of ASIC and System, School of Microelectronics, Fudan University, Shanghai 200433, China

[3] Institut für Materialphysik; Westfälische Wilhelms-Universität Münster, Wilhelm-Klemm-Straße 10, 48149, Münster, Germany

[4]Department of Physics, University of Münster, 48149 Münster, Germany

*Corresponding author: E-mail: harish.bhaskaran@materials.ox.ac.uk

**Keywords:** Phase change materials, Antimony, Ultrafast switching, Metallic glass, Femtosecond Processing

**S1. Experimental Setup**

**S2.Switching Speed Measurements**

**S3.Sb Length Dependence on contrast**

**S4.Effect of Capping Layer**

**S5.Pulse Energy calculations**

**S6. Sub-millisecond Readout**

**S7. Multilevel Readout stability**

**S1.Experimental Setup**

The experimental setup includes two optical lines, as shown in Figure S1, in Red and Blue corresponding to Pump and Probe line. For the Pump line, a fibre coupled femtosecond laser, centred at 1560nm wavelength, with pulse width of 800fs and repletion rate of 40 MHz is used. To select a single or train of pulses, this signal is passed through a home-built pulse picker comprising of AOM. The amplitude of the pulses is modulated using an EOM and amplified using a high peak power Erodium doped fibre amplifier (EDFA). The probe signal is a continuous laser centred at wavelength 1580. Both the Pump and probe signal are polarization controlled to get maximum transmission through the device. The two signals are injected in and out of the device using different grating couplers. The output signal from the probe line is passed through an optical filter to block the signal from Pump and is used to monitor the change in transmission upon switching

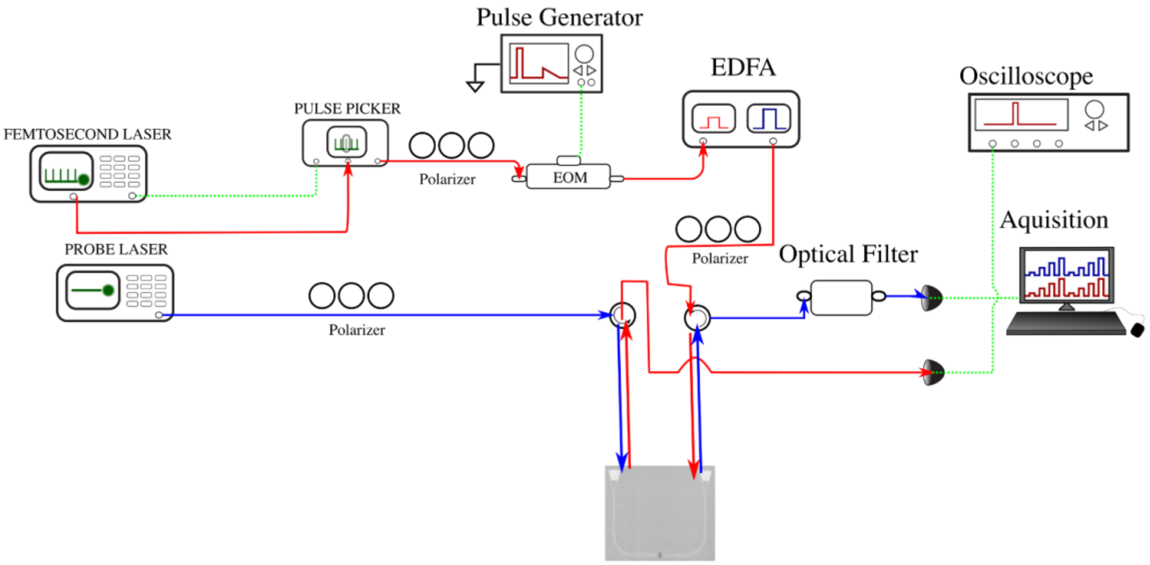


**Figure S 1** : Experimental setup used to carry out the switching experiments. The setup comprises of two optical lines ( Pump and Probe) depicted in Red and Blue respectively and electrical signal lines depicted in green with arrows pointing the direction of beam propogation.

**S2.Switching Speed Measurements**

To calculate the switching speed of Sb on waveguide, we perform time resolved switching experiments using a 4 port device as shown in Figure S2 (a). Probe signal output is connected to a 125 MHz photodetector to record the change in transmission on sending a Pump pulse. A single write pulse is used to switch the material from crystalline state to amorphous state. Due to low signal to noise ratio , we average the change for a set of 10 reading and find a rise time (from 10% of change to 90%) of 2ns , which corresponds to operation speed of up to 500 MHz ( Figure S2 (b)). The experiments are repeated for different pump power to achieve different memory levels using single sub picosecond pulse of energy P1-P4, with P1>P4.

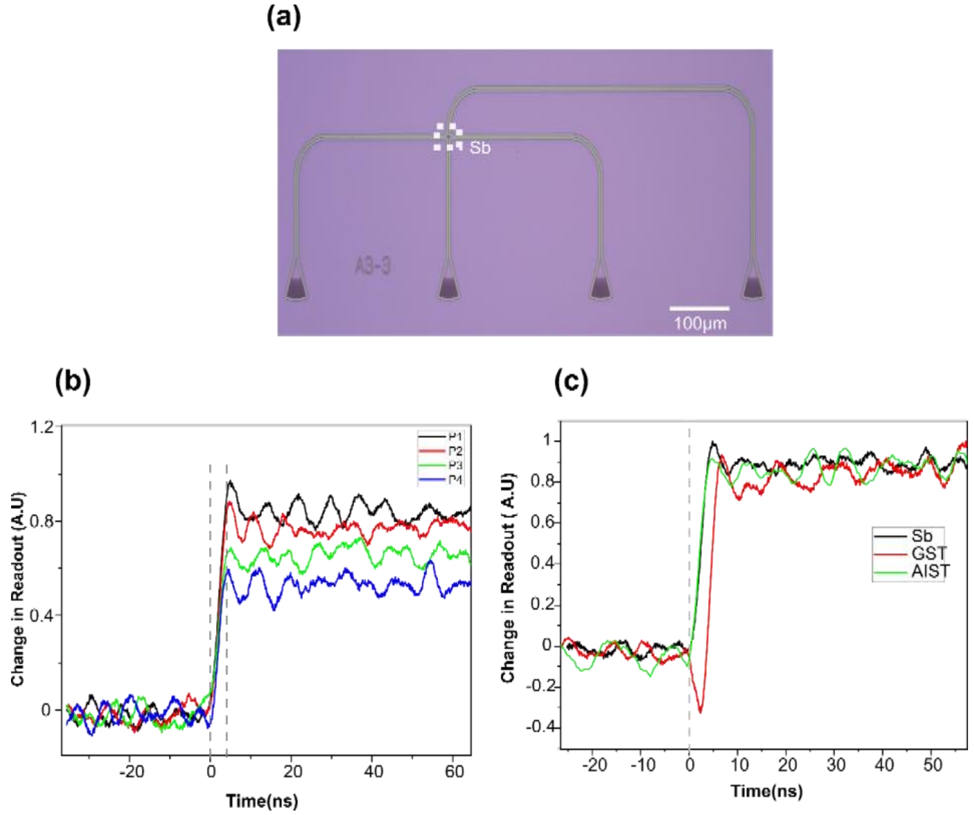


**Figure S 2** (a) Optical image of 4 port device used to measure the switching speed. Phase change material is deposited on the optical crossing. Using one of the ports as input for pulse and other as probe, time resolved switching characteristics is obtained. (b) Time resolved switching dynamics of Sb, resulting in a rise time of 2ns for a single sub picosecond pulse, on switching from crystalline to amorphous state. (c) Experimental comparison of switching speed of Sb is with other known phase change materials like GST and AIST using a single sub picosecond pulse for amorphisation.

Furthermore, the switching speed of Sb was compared with other well-known phase change material using the pump-probe technique. The switching speeds of Sb, AIST and GST

are on same timescales as shown in Figure S2(c). However, as shown in our previous work[23], a very short femtosecond pulse is enough to amorphise Sb, therefore the current switching speed for Sb is limited only by photonic system rather than material itself.

**S3.Sb Length Dependence on contrast**

Experimental results showing the effect of length of Sb on the loss in amorphous and crystalline state. Different lengths of Sb were deposited on a waveguide and the transmission of the as deposited Sb on waveguide was noted. The experiments were repeated after annealing the samples on a hot plate at 230 °C for 5 minutes to completely crystallize Sb. Increasing the length of Sb results in a higher absorption and hence a larger contrast ( Figure S3).

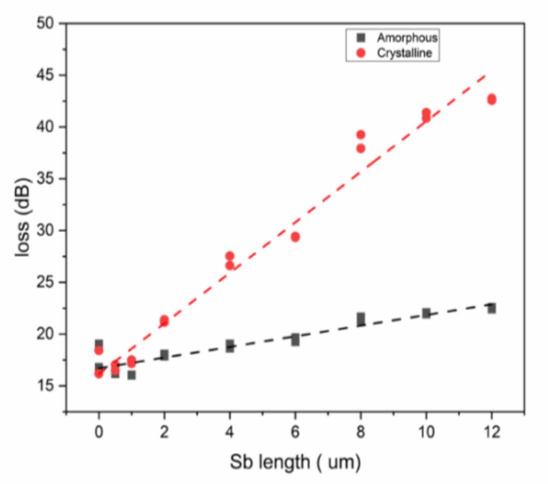


**Figure S 3**: Experimental results showing the effect of increasing Sb length on a waveguide on the loss (absorption of light) in as deposited amorphous and hot plate annealed crystalline state. Increasing the length of Sb results in higher contrast between amorphous and crystalline states.

**S4.Effect of Capping Layer**

Further to our experiments with uncapped antimony thin films. We studied the performance of our device with an additional 10nm capping layer of Indium tin oxide (ITO). Due to an increased absorption of light due to the capping layer, a 4 µm long device is enough to get a 10% change in contrast as compared to 10µm long uncapped device ( Figure S4(a)). Further we investigated Raman spectra of both capped and uncapped crystalline

antimony, after annealing in air at 230 °C. No antimony oxide (Sb2O3) peaks at 191cm$^{-1}$ and 255 cm$^{-1}$ were observed in our samples ( Figure S4 (b)).

**S5.Pulse Energy calculations**

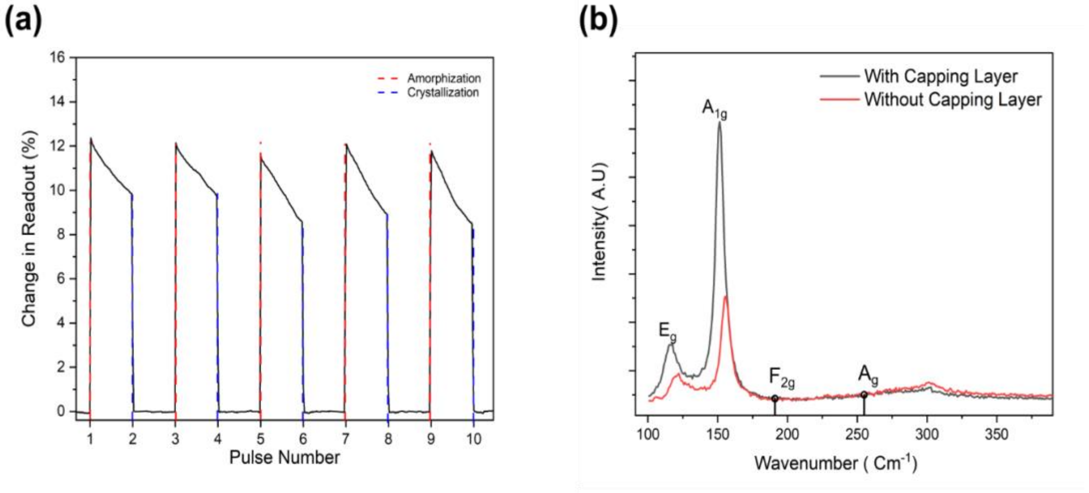


**Figure S 4** : a) Binary switching a 4 µm long of Sb with 10nm ITO capping on the using single, high energy (194 ±35pJ) femtosecond pulse (800fs) – (amorphization pulse, red dashed vertical lines indicate this pulse). 100 low energy (45±9pJ) pulses (Crystallization Pulse, blue dashed lines indicate this pulse sequence) crystallize the sample. b) Raman spectra of capped and uncapped crystallized thin film of Sb on waveguide with their $E_g$ and $A_{1g}$ vibration modes. The circles denote the typical $F_{2g}$ and $A_g$ modes of $Sb_2O_3$, clearly showing absence of any antimony oxide in our samples.

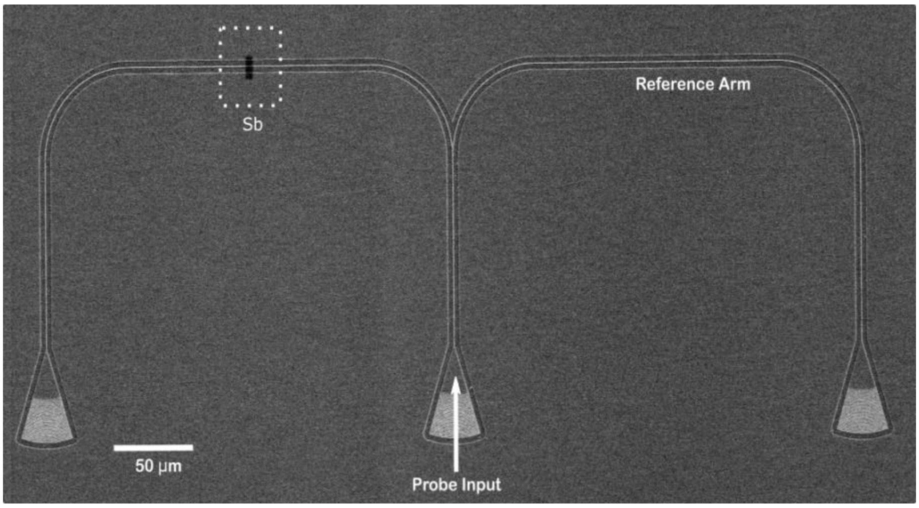


**Figure S 5:** SEM image of the device used to for long term stability experiment. The probe signal is split using a Y- splitter into two arms one arm used to measure the transmittance through Sb and another reference arm to monitor the input power fluctuations and drift in optical signal. The signal from reference arm is used to normalize the transmission from Sb arm and calculate the retention time of switched Sb.

To calculate the energy we use a Pyroelectric sensor from ‘Ophir’, which has an energy measurement resolution of few Nano joule to hundreds of Nano joule, with a maximum repetition rate of 15 kHz. To accurately measure the energy of a single pulse we send train of 240-800 pulses and corresponding energies are recorded. This is used to estimate energy of single pulse.  Each train of pulse is sent 100 times. The average and the standard deviation obtained is used to report the switching energy and the error associated.

**S6. Sub-millisecond Readout**

We characterise the stability and repeatability of achieving the binary memory levels in sub millisecond range. 10 μm Sb device is amorphized using single fs pulse and subsequently crystallized using 100 low energy fs pulses after 100 μs. The change in transmission is recorded using an oscilloscope Figure S6(a). The switching is repeated multiple time and

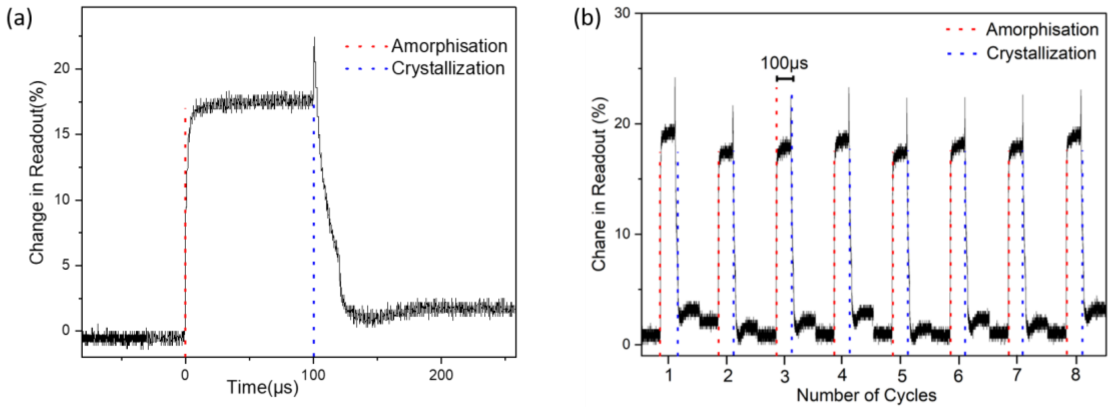


**Figure S 6:** (a) Sub-millisecond readout for binary switching of 10 μm long of Sb. (b) Experiments at in (a) repeated multiple time, with less than 2% variation is memory levels.

shows a repeatability with less than 2% variation, multiple plots are stitched together and presented in  Figure S6 (b).

**S7. Multilevel Readout stability**

We characterise the stability and repeatability of achieving the 4 intermediate memory levels in millisecond range. 10 μm Sb device is amorphized using single fs pulse and subsequently crystallized using 100 low energy fs pulses. The change in transmission is recorded using an

oscilloscope for different energy of amorphisation pulses (Figure S7). We observe stability of intermediate memory over 100ms with variation readout of 1%.

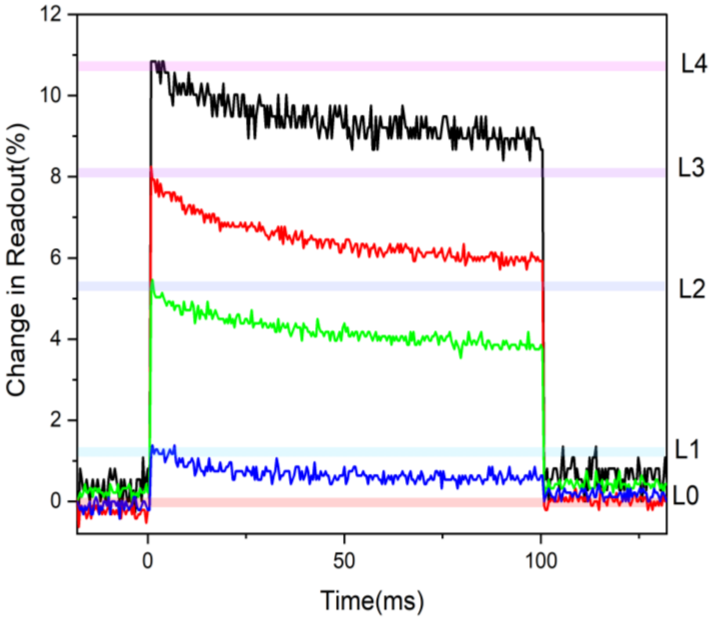


**Figure S 7:** Millisecond readout for intermediate memory levels obtained for different amorphisation pulse energies. Memory levels show variation of 1% in readout over 100ms time duration.
